# Age‐related Changes with the Trabecular Bone of Ward's Triangle and Neck‐shaft Angle in the Proximal Femur: A Radiographic Study

**DOI:** 10.1111/os.13923

**Published:** 2023-10-19

**Authors:** Kai Ding, Yanbin Zhu, Jiaxing Li, Peizhi Yuwen, Weijie Yang, Yifan Zhang, Haicheng Wang, Chuan Ren, Wei Chen, Qi Zhang, Yingze Zhang

**Affiliations:** ^1^ Department of Orthopaedic Surgery, Hebei Orthopaedic Clinical Research Center The Third Hospital of Hebei Medical University Shijiazhuang China; ^2^ Key Laboratory of Biomechanics of Hebei Province Orthopaedic Research Institute of Hebei Province Hebei China; ^3^ NHC Key Laboratory of Intelligent Orthopaedic Equipment (The Third Hospital of Hebei Medical University) Shijiazhuang China; ^4^ Engineering Research Center of Orthopaedic Minimally Invasive Intelligent Equipment Ministry of Education Shijiazhuang China; ^5^ Chinese Academy of Engineering Bingjiaokou Hutong Bejing China

**Keywords:** Compression trabeculae, Neck‐shaft angle, Proximal femur, Tension trabeculae, Ward's triangle

## Abstract

**Objective:**

The Ward triangle is an important area used clinically to diagnose and assess osteoporosis and its fracture risk in the proximal femur. The main objective of this study was to investigate the rules of development and maturation of the trabeculae of Ward's triangle to provide a basis for the prevention and treatment proximal femur fracture.

**Methods:**

From January 2018 to December 2019, individuals from 4 months to 19 years old who underwent hip growth and development assessments at the Third Hospital of Hebei Medical University were selected retrospectively. The outpatient electronic medical record system was used to collect information such as age, gender, imaging images, and clinical diagnosis. The development score and maturity characteristics of the trabecular bone were analyzed using hip radiograph data. Correlation analysis was performed to identify the relationship among age, neck‐shaft angle and development and maturity score of the trabecular bone.

**Results:**

A total of 941 patients were enrolled in this study, including 539 males and 402 females. Primary compression trabeculae were all present at 1 year of age and matured at 7 years of age and older; primary tension trabeculae were all present at 4 years of age and matured at 18 years of age. Secondary compression trabeculae were present at 4 years of age and matured at 18 years of age. In addition, the neck‐shaft angle progressively decreases from 4 months to 14 years of age but barely changes between 15 and 19 years of age.

**Conclusion:**

In short, the development and maturation of the trabeculae in the ward’ triangle followed a specific temporal pattern that was related to the neck‐shaft angle. Therefore, these findings can help us understand structure and mechanical characteristics of proximal femoral trabeculae, and improve our understanding of the mechanism and treatment of proximal femoral fractures.

## Graphical abstract

This study aimed to identify age‐related changes with the trabecular bone of Ward's triangle and neck‐shaft angle in the proximal femur, to quantitatively determine the rules of development and maturation of the trabeculae of Ward's triangle, so as to guide clinical treatment.

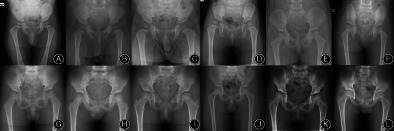

## Introduction

The number of hip fractures is expected to reach 6.3 million by 2050, with osteoporosis accounting for 80%.^1^, ^2^ Hip osteoporosis is characterized by reduced trabecular bone mass and Ward's triangle bone quality. Degeneration of trabecular structure in Ward's triangle increases hip fracture risk and secondary fracture which poses many serious medical challenges that seriously threaten the health and life expectancy of the elderly population.^3^, ^4^, ^5^, ^6^, ^7^, ^8^, ^9^

The proximal femur developed a special system of medial and lateral trabeculae as humans progressed from crawling to standing, allowing it to respond to tension and compression forces of proximal femur.^10^ The most distinguishing trabeculae in humans are those of the proximal femur, specifically the primary compression, primary tension, and secondary compression trabeculae, which intersect to form “Ward's triangle.” A recent μCT‐based analysis revealed trabecular bone bears 40%–70% of the mechanical load in the femoral neck.^11^ The trabeculae breakage of Ward's triangle plays a critical “initiating role” in the development of proximal femoral fractures.^12^, ^13^, ^14^ Nawathe *et al*.^15^ concluded that only about 1.5%–6.4% of ward's trabeculae were found to be initially ruptured, resulting in proximal femoral fractures, in lateral impact falls. Therefore, Ward's triangular trabeculae are the primary tissue for assessing osteoporosis in the Singh index and fracture risk.^16^, ^17^

Due to its mechanical characteristics and special structure, there are currently many studies on Ward's triangle trabeculae from macroscopic and microscopic levels, including bone tissue, imaging examination, and biomechanical study, and so forth.^18^, ^19^, ^20^ The research mainly focuses on the law of osteoporosis, but ignores the law of trabecular development and maturation. In addition, the evaluation criteria of development and maturation have been lacking, resulting in confusing definition of trabecular bone development. The study of developmental rules helps us not only to assess hip development in children, but also to gain insight into the mechanism of fracture. In view of the above, this study aims to: (i) investigate age‐related change in the neck‐shaft angle and development and maturation of trabeculae; (ii) establish criteria for trabecular bone growth and maturation; and (iii) analyze the relationship between developmental and maturation of trabeculae in Ward‘s triangle and occurrence and treatment of hip fractures.

## Materials and Methods

### Clinical Data

This was a retrospective study using the outpatient electronic medical record system the Third Hospital of Hebei Medical University in China. Between January 2018 and December 2019, patients aged 4 months to 19 years were included in this study. The inclusion criteria were: (i) individuals from 4 months to 19 years old; (ii) normal growth and development of proximal femur; and (iii) complete outpatient data. Exclusion criteria were: (i) out of age range; (ii) patients with abnormal hip growth and development; and (iii) hip osteoporosis; fractures.

A total of 951 patients were assessed for hip growth and development. In this study, demographic data of the patient population, including diagnosis and hip X‐ray films, were collected and recorded from the outpatient care system. Once patients were enrolled in the study, their guardians were contacted to consent to the possible use of imaging for clinical research. All data were analyzed anonymously to protect patient privacy. This study was approved by the Ethics Committee of the Third Hospital of Hebei Medical University (2014‐015‐1).

### Femoral Imaging Measurements

The primary exposure variable in this study was age. To observe the progression of the earliest appearance of trabeculae in children under 1 year of age, we divided the children into three groups: those aged 4–6 months, 7–9 months, and 10–12 months.

The femur images included in this study were a plain film of both hip joints, which were used to assess bone morphometry and trabecular differentiation and maturation in individuals aged 4 months to 19 years. The neck‐shaft angle (the angle of intersection between the central axis of the femoral neck and the femoral shaft) was also measured. Many data points were analyzed to determine the appropriate time point for trabecular differentiation and maturation. A trabecular differentiation and maturation table was developed to diagnose the proximal femur trabecular bone. Based on the earliest time point of appearance and mature of trabecular bone, this study divided the development process into three stages: no appearance (there were no regular bundles of trabeculae on the hip radiograph), not fully mature (a state between non‐ appearance and full mature on the hip radiograph), and fully mature (adult hip trabecular bone imaging was defined as fully mature). All radiological parameters were measured and scored by two orthopedic surgeons using our PACS (medSynapse version 5.0.1.3) with the exception of the femoral neck‐shaft angle and trabecular maturation score. For a fair and comparable assessment, a third observer was selected when there were differences between the two.

### Scoring Criteria

**FIGURE 1:**
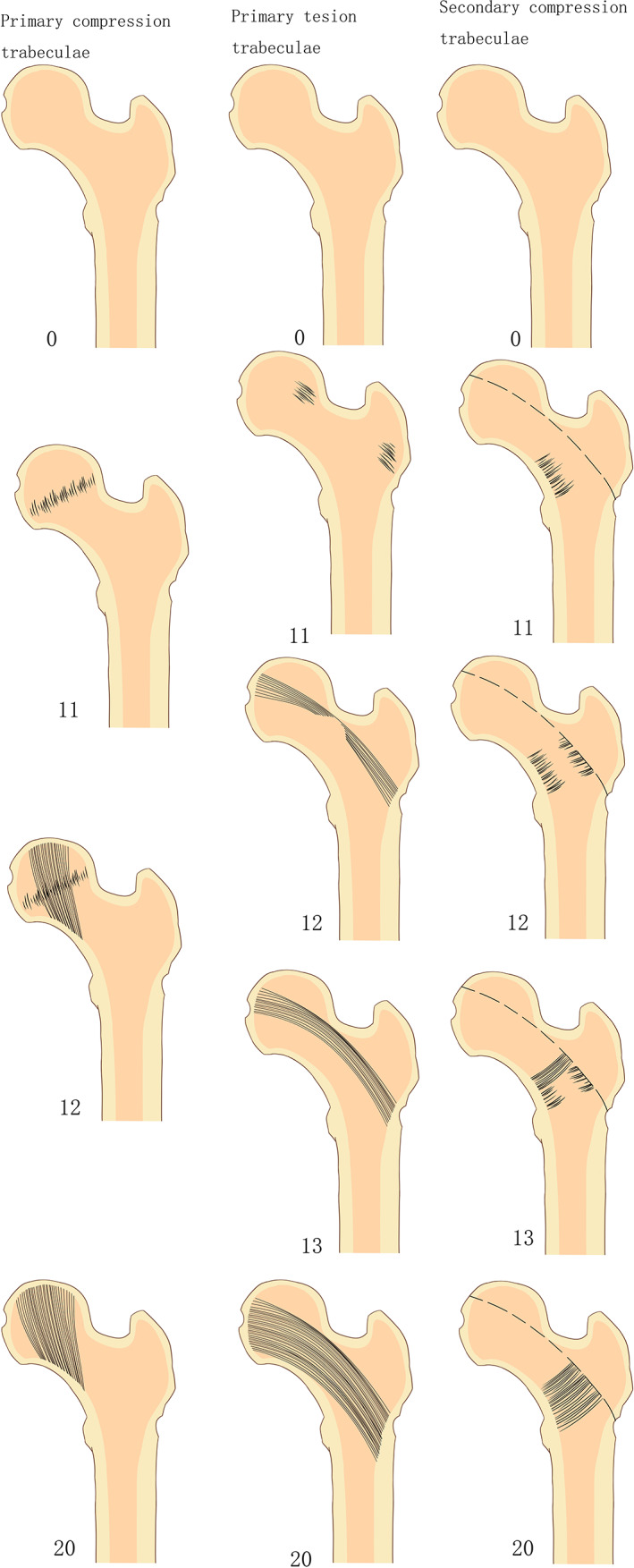
The pictures of developmental maturation scoring of the trabeculae bone in ward triangle.

Time points for trabecular appearance and maturation were defined in this study as regular bundles of trabeculae and trabecular meshwork, respectively, with normal bone mineral density. The criteria classify the trabecular development process as non‐emerging, partially mature, and fully mature stages according to the trabecular emergence and maturation nodes. Partial maturation means just emerging, incomplete trabecular but intact length, and incomplete trabecular but intact density. Based on the Singh criteria,^18^, ^21^ the trabecular maturation and development score was constructed as follows (Table 1; Figure 1). Primary compression trabeculae: 0 for no trabecular bone, 11 for trabecular bone arising from partial trabecular bone proximal femur, 12 for the length and density reached maturity, and 20 for mature trabecular bone. Primary tension trabeculae: 0 for no trabecular bone, 11 for emerging trabecular bone but low length and density, 12 for trabecular bone with certain some mature density but poor length, 13 for trabecular bone having some mature length but poor density and 20 for mature trabecular bone. Secondary compression trabeculae: 0 for no trabecular bone, 11 for emerging trabecular bone but low length and density, 12 for trabecular bone with a specific segment of mature density but poor length, 13 for trabecular bone with some mature length but poor density, and 20 for mature trabecular bone. (Table 1; Figure 1).

**TABLE 1:** Scoring criteria for each trabecular bone in ward's triangle

Primary tension trabeculae		Primary compression trabeculae		Secondary tension trabeculae	
Score	Definition	Score	Definition	Score	Definition
0	Bone trabeculae were not present	0	Bone trabeculae were not present	0	Bone trabeculae were not present
11	Bone trabeculae appeared	11	Bone trabeculae appeared	11	Bone trabeculae appeared
12	The length and density partial trabecular bone reached maturity	12	The density of partial trabecular bone reached mature density but the length was poor	12	The density of partial trabecular bone reached mature density but the length was poor
		13	The length of partial trabecular bone reached maturity but the density was poor	13	The length of partial trabecular bone reached maturity but the density was poor
20	The trabecular bone reached maturity	20	The trabecular bone reached maturity	20	The trabecular bone reached maturity

### Statistical Analysis

Statistical software SPSS 25.0 (SPSS, Chicago, IL, USA) and Origin 2021 (OriginLab, Northampton, MA, USA) was used for data processing and statistical analysis. Continuous data with a normal distribution were expressed as mean ± standard deviation. The inter‐observer comparisons were analyzed using consistency test. *T*‐test was used to evaluate the between sex‐related neck‐shaft angle difference. Categorical data were statistically analyzed using Pearson's correlation coefficient or the Spearman correlation coefficient. consistency test to the inter‐observer comparisons. *p* < 0.05 was considered statistically significant.

## Results

A total of 941 eligible individuals, including 539 male young children and 402 female young children, were included according to the inclusion and exclusion criteria from January 2018 to December 2019 in the outpatient system (Table 2).

**TABLE 2:** The number of included children of corresponding age group

Age	Number of cases
m 4	72
m 7	57
m 10	36
1 y	39
2 y	80
3 y	61
4 y	41
5 y	46
6 y	41
7 y	27
8 y	56
9 y	57
10 y	49
11 y	55
12 y	62
13 y	43
14 y	28
15 y	16
16 y	29
17 y	18
18 y	15
19 y	13
Total	941

### Aged‐related Change in the Neck‐Shaft Angle

**FIGURE 2:**
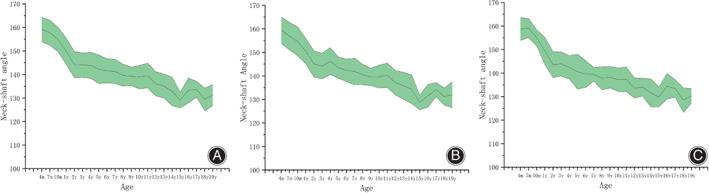
Relationship between neck‐shaft angle and age. (A), The neck‐shaft angle of the included individual; (B), The femoral neck‐shaft angle of male young children; (C), The neck‐shaft angle of female young children.

The neck‐shaft angle decreases with increasing age. And the neck‐shaft angle decreased significantly from 4 months to 14 years of age (Pearson's correlation coefficient: *r* = −0.760; *p* < 0.001). However, there was no significant change from 14 to 19 years of age (Pearson's correlation coefficient: *r* = −0.098; *p* = 0.376). The femoral neck‐shaft angle of male children was higher than that of female children at the age of 4, 8 and 12 years. The intraclass correlation coefficient (ICC) value of the two observers was 0.84 (*p* < 0.001), and the repeatability was high. (Table 3; Figure 2).

**TABLE 3:** Aged‐related change in the neck‐shaft angle

Age	Total (Mean ± SD)	Male (Mean ± SD)	Female (Mean ± SD)	*F*	*p* value
m 4	159.08 ± 5.19	159.42 ± 5.56	158.75 ± 4.85	0.542	0.589
m 7	157.75 ± 5.29	156.86 ± 5.83	159.16 ± 4.03	−1.761	0.084
m 10	154.78 ± 5.16	154.68 ± 6.11	154.93 ± 3.40	−0.155	0.877
1 y	149.91 ± 5.55	150.56 ± 5.38	149.41 ± 5.75	0.637	0.528
2 y	144.18 ± 5.55	145.08 ± 5.58	143.57 ± 5.51	1.192	0.237
3 y	144.01 ± 5.22	144.15 ± 5.43	143.87 ± 5.09	0.207	0.837
4 y	143.89 ± 5.52	146.25 ± 5.68	142.38 ± 4.96	2.304	0.027*
5 y	142.23 ± 6.04	143.48 ± 4.51	140.60 ± 7.40	1.536	0.135
6 y	141.55 ± 5.13	142.36 ± 4.74	139.58 ± 5.71	1.609	0.116
7 y	141.26 ± 5.12	141.88 ± 5.64	139.50 ± 2.83	1.436	0.165
8 y	139.68 ± 4.65	140.50 ± 4.34	137.79 ± 4.91	2.063	0.044*
9 y	139.218 ± 3.92	139.56 ± 3.67	138.23 ± 4.57	1.127	0.265
10 y	138.88 ± 4.99	139.68 ± 4.85	137.22 ± 5.01	1.650	0.106
11 y	139.52 ± 5.25	140.27 ± 5.13	137.32 ± 5.15	1.854	0.069
12 y	136.05 ± 5.14	137.12 ± 5.17	133.63 ± 4.27	2.573	0.013*
13 y	135.10 ± 5.07	135.81 ± 5.72	134.03 ± 3.77	1.129	0.266
14 y	132.78 ± 6.09	134.31 ± 6.06	131.58 ± 5.97	1.358	0.183
15 y	129.13 ± 3.31	128.70 ± 3.13	129.83 ± 4.19	−0.441	0.675
16 y	133.21 ± 5.28	131.54 ± 4.84	134.38 ± 5.40	−1.454	0.157
17 y	133.72 ± 3.37	134.14 ± 3.02	133.45 ± 3.70	0.411	0.686
18 y	129.40 ± 4.78	131.20 ± 3.63	128.50 ± 5.19	1.034	0.320
19 y	131.23 ± 4.48	132 ± 5.54	130.33 ± 3.08	0.653	0.527

*Note*: * *P*<0.05; “*F* and *p* value” for value and significance of differences in neck shaft angle between genders.

### Sex‐related Differences in Neck‐shaft Angle

The neck‐shaft angle decreases with increasing age for male and female children. The neck‐shaft angle of male children was greater than that of female children between the ages of 1–14 years; there was a statistically significant difference in neck angle between male and female children at the ages of 4, 8, and 12 years, and no statistically significant difference at the other ages. There was no significant difference in the neck‐shaft angle between boys and girls within 1 year of age and between 14 and 19 years of age.

### Aged‐related Development and Maturation of Bone Trabeculae

**FIGURE 3:**
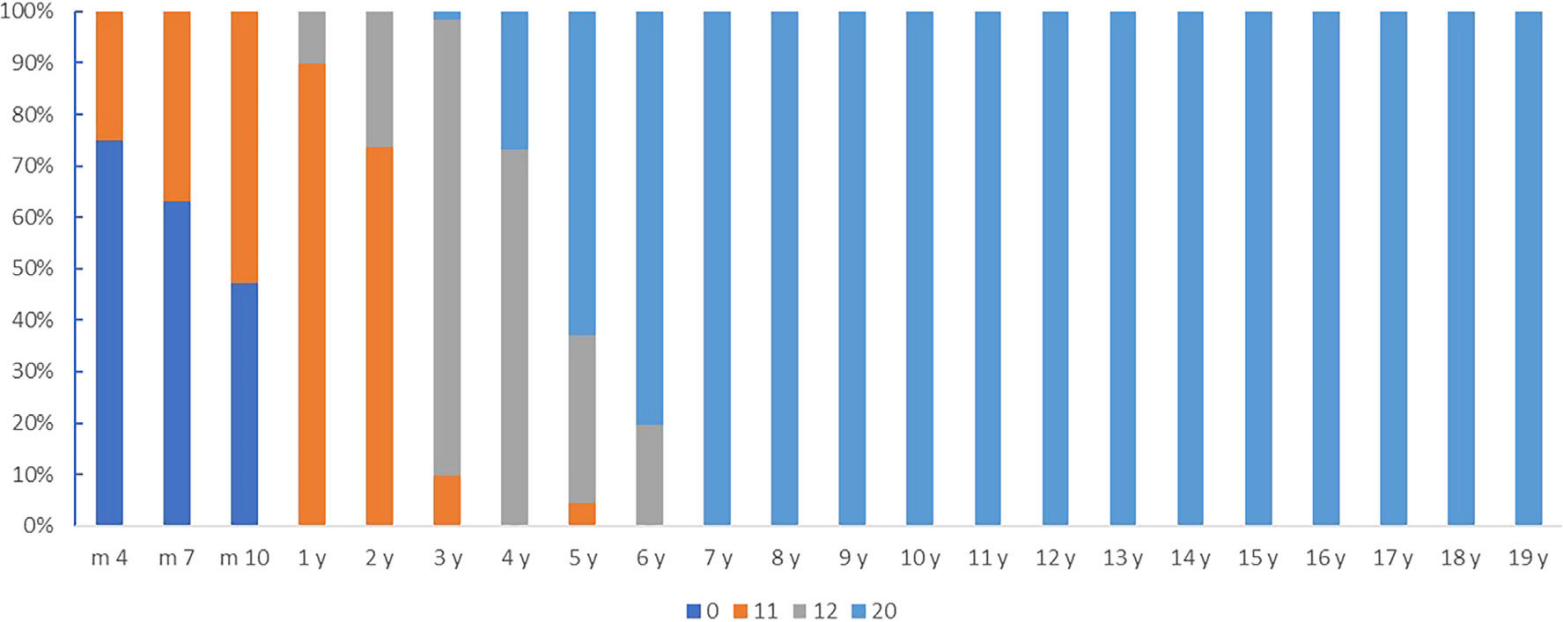
Constituent ratios of primary compression trabecular development and maturation at different ages.

Primary compression, primary tension and secondary compression are the most characteristic trabeculae in the proximal femoral trabeculae. In terms of the earliest time of appearance of trabeculae, the results showed that compared with primary tension and secondary compression, primary compression trabeculae were found in children aged 4 months. Infants aged 4–6 months had 15.28% (18/72), children aged 7–9 months had 22.81% (21/57) and older children aged 10–12 months had 44.44% (19/36). In children aged 1 year and older, the primary compression trabeculae were found in all included children, and the trabecular development scores increased with increasing age. Up to 7 years of age and above, the trabecular development score is stable for all children and is the highest score (20 points) (Figure 3). The kappa value of the two observers' evaluation of development and maturation of primary compression trabeculae was 0.903 (*p* < 0.001), and the repeatability was high.

**FIGURE 4:**
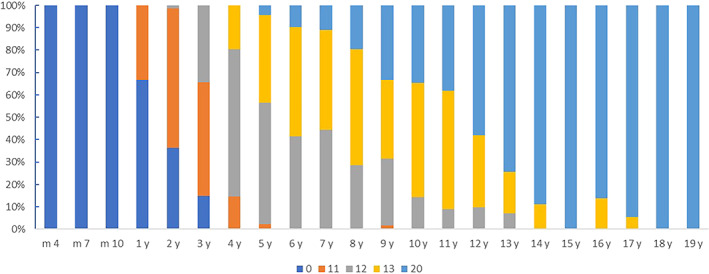
Constituent ratios of primary tension trabecular development and maturation at different ages.

However, the percentage of primary tension or secondary compression was 0.00% in children less than 1 year old. In children aged 4 years and older, all primary tension trabeculae were found. And in children aged 6 years and older, all the secondary compression trabeculae were found. Up to the age of 18 years and above, the trabecular development scores and the percentage of individuals with both are shown in Figure 4. Primary compression trabeculae develop and mature gradually with increasing age (Spearman correlation coefficient: *r* = 0.881; *p* < 0.001). The kappa value of the two observers' evaluation of development and maturation of primary tension trabeculae was 0.856 (*p* < 0.001), and the repeatability was high.

**FIGURE 5:**
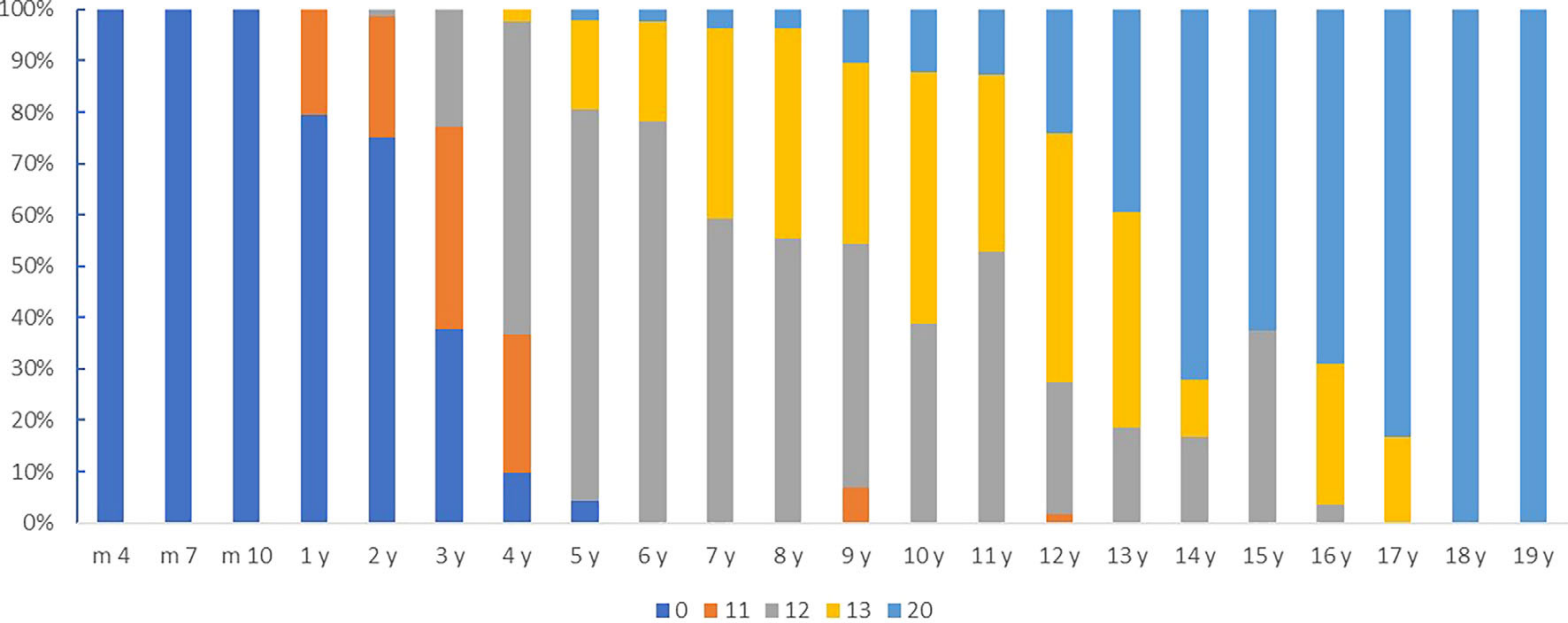
Constituent ratios of secondary compression trabecular development and maturation at different ages.

**FIGURE 6:**
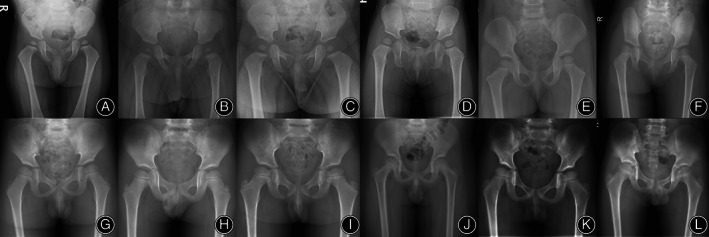
Hip radiographs of children aged from 4 months to 9 years, the femoral neck‐shaft angle decreases with the increase of age. (A): 6 months old; (B): 7 months old; (C): 10 months old; (D): 1 year old; (E): 2 years old; (F): 3 years old; (G): 4 years old; (H): 5 years old; (I): 6 years old; (J): 7 years old. (K): 8 years old; (L): 9 years old.

**FIGURE 7:**
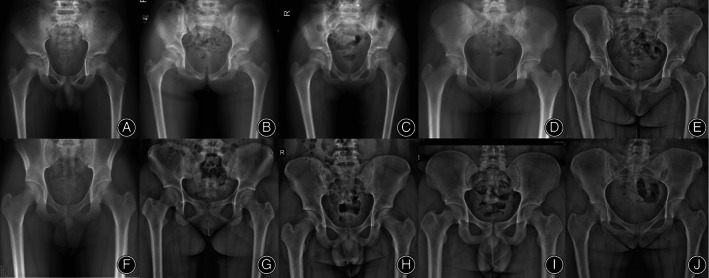
Hip radiographs of children aged 10–19 years, the femoral neck‐shaft angle decreases with the increase of age. (A): 10 years old; (B): 11 years old; (C): 12 years old; (D): 13 years old; (E): 14 years old; (F): 15 years old; (G): 16 years old; (H): 17 years old; (I): 18 years old; (J): 19 years old.

According to the maturation time of the trabeculae, the maturation of primary tension trabeculae first appeared at the age of 3 years and was fully mature in children aged 7 years and older. Primary tension trabeculae first matured at the age of 5 years and were fully mature at the age of 18 years and older (Figure 5). Primary tension trabeculae develop and mature gradually with increasing age (Spearman correlation coefficient: *r* = 0.900; *p* < 0.001). Maturation of secondary compression trabeculae occurs as early as 5 years of age and is fully mature in children 18 years of age and older (Figures 6 and 7). Secondary compression trabeculae develop and mature gradually with increasing age (Spearman correlation coefficient: *r* = 0.881; *p* < 0.001). The kappa value of the two observers' evaluation of development and maturation of secondary compression trabeculae was 0.918 (*p* < 0.001), and the repeatability was high.

### Correlation Analysis between Trabeculae of Ward's Trabeculae and Neck‐shaft Angle

Primary compression, primary tension and secondary compression trabeculae gradually develop and mature with decreasing neck‐shaft angle. Spearman correlation coefficient was as follows: primary compression trabeculae (*r* = −0.716; *p* < 0.001); primary tension trabeculae (*r* = −0.770; *p* < 0.001); and secondary compression trabeculae (*r* = −0.753; *p* < 0.001).

## Discussion

Using hip X‐ray films, we investigated the development and maturation of trabecular bone and the change of neck‐shaft angle in children to better understand the degeneration law of hip trabeculae. Based on our studies, the neck‐shaft angles gradually decreased from 4 months to 14 years of age, and they did not change significantly from 14 to 19 years of age. Furthermore, bone trabeculae in Ward's triangle are closely related to lower limb stress conduction and proximal femoral fracture. Additionally, tension has a slower effect on bone growth than compression. We would explain our findings from the following aspects, for example advantages and disadvantages of the scoring criteria, developmental and maturation rules of trabecular bone and its relation to neck shaft angle.

### Developmental and Maturation Patterns of Trabecular Bone in Ward's Triangle and Its Relation to Neck Shaft Angle

This study has many important implications. The time of earliest appearance of primary compression, primary tension and secondary compression trabeculae was 4 months, 1 and 2 years respectively. The maturation time span of primary compression, primary tension and secondary compression trabeculae were 7, 16 and 16 years, respectively. The development and maturation of the primary compression trabeculae is significantly faster than that of the primary tension and secondary compression trabeculae. We believe that this is closely related to the growth characteristics and stress conduction of the trabeculae. Tension has a slower effect on trabecular development than compression.

The primary compression trabeculae are the main bone components with the shortest distance to resist human loading, while the primary tension and secondary compression trabeculae play an auxiliary role in the proximal femur. Additionally, these results are consistent with the mechanisms responsible for the development of osteoporosis, in which the trabecular structure of the non‐primary stress area weakens first at age.^22^ Moreover, there was a negative linear correlation between the neck‐shaft angle and development and maturation of trabecular bone. The neck‐shaft angle transmits the compressive and tension forces on the femoral head more perpendicularly to the femoral neck area.^23^ And the mechanical conduction could change the relative density, cell wall properties and cell geometry of bone trabeculae.^24^ And the compression and tension load in the proximal femur increases as the neck‐shaft angle decreases. Therefore, the reduction of the neck‐shaft angle and development of trabecular bone are important representation of deformation and structural changes under loading stress. Furthermore, the maturation of the secondary compression trabeculae is accompanied by the rapid maturation of the primary tension trabeculae.

There is an apparent correlation between the maturation of the secondary compression trabeculae and their structural and distributional characteristics. The primary compression trabeculae and the assertive trabeculae form a triangular cantilever beam structure against human loads. Two trabeculae, particularly the primary tension trabeculae, showed different varus patterns when in the standing position. Secondary compression trabeculae must perform support tension trabeculae to prevent varus differences. Therefore, the presence of secondary compression trabeculae is beneficial in supporting the stability of the proximal femoral trabecular system. Finally, the neck‐shaft angle changed more rapidly than the development of primary tension trabeculae. This is due to the fact that the development of bone and trabecular bone is not consistent. The development of tension trabeculae is characterized by the increase of local trabecular density, the presence of bone trabecular bundles and the maturation of trabecular bone. The maturation of trabecular bone requires a long period of loading.

### Relationship between the Developmental and Maturation Patterns of Trabecular Bone in the Ward Triangle and the Occurrence and Treatment of Hip Fractures

The development and maturation rules of trabecular bone have important implications for the occurrence and treatment of proximal femoral fractures. First, the strength of the greater trochanter can influence the initial position of the proximal femoral fracture. According to the mechanism and structural characteristics of the injury, the greater trochanter is the predominant site of low‐energy injury leading to proximal femoral fractures in older people who fall to the ground.^25^ According to our results, the epiphysis of the greater trochanter gradually developed and fused with the primary tension trabecula to form an arched structure similar to the “percussion point” structure. A proximal femur fracture is 3–5 times more likely in a lateral fall than in a forward, backward, or vertical downward fall and over 30 times more likely if the greater trochanter is involved in a lateral fall.^26^, ^27^, ^28^ The upper lateral femoral neck is a transitional structure between the femoral head and the greater trochanter, and its morphological changes are significantly greater than those of other parts. Therefore, the greater trochanter has a high structural strength, and the upper and lateral of the femoral neck has the greatest strain under the external force, leading to a high incidence of femoral neck fracture.^11^, ^29^, ^30^ On the contrary, the greater trochanter has a weak structure, and the bone is ruptured first, resulting in an intertrochanteric fracture. Second, the current design of internal fixation for proximal femoral fractures is based on restoring resistance to compression, including screw increases and structural modifications. However, the complication rate is still high.^31^, ^32^, ^33^, ^34^ The development time of the primary tension trabeculae is longer and the structural reconstruction is slower, which is different from the current design idea of internal fixation. As a result, the design of tension resistance is ignored. The presence of tension delays or hinders trabecular healing and ultimately leads to complications. Based on the characteristics of the proximal femoral trabecular structure, Ding *et al*.,^13^, ^14^ and Wang et al.^35^ introduced the concept of stable triangular structure into the design of internal fixators and developed a triangular‐support internal fixation system based on the theory of triangular‐support fixation. The results showed that the fixation system could effectively improve the structural stability and stress distribution of internal fixation of intertrochanteric fractures compared with gamma nails, DHS and PFNA. These results also confirmed that the morphology and structure of the trabecular bone can adapt to the function of the hip joint. Therefore, the future direction in the treatment of proximal femoral fractures will be to develop internal fixation devices that better match the biomechanical characteristics of the trabecular bone.

### Advantages and Disadvantages of the Scoring Criteria

Due to the limited research methods and lack of research literature, this study used anteroposterior hip radiographs to semi‐quantitatively assess the development and maturation of trabecular bone. Based on the Singh criteria for hip osteoporosis, this study establishes diagnostic staging criteria for trabecular bone growth and maturation.^21^ These criteria allow us to study the temporal patterns and structural characteristics of different types of trabeculae. In addition, the scoring table proposed in this study has easy access to data, more intuitive results, short learning curve, and good inter‐observer consistency. However, the criteria have not been tested for validity and accuracy. The development and maturation of trabecular bone can be studied in detail in the future using further research methods and techniques.

### Limitations

This study has some limitations. First, the results may be biased due to the single‐center nature and the limited number of subjects included. To our knowledge, there are no similar studies about evaluation criteria of development and maturation of ward triangle trabeculae. The criteria for trabecular maturation used in this study need to be validated by more studies and its reliability needs further verification. Finally, the youngest population included was 4 months of age, as growth and development in children under 4 months of age was mainly assessed by ultrasound. Therefore, primary compression trabeculae may appear earlier than 4 months of age.

## Conclusion

In conclusion, the neck shaft angle decreases as well as Ward's trabeculae mature with age. In addition, the primary compression trabeculae develops significantly earlier than primary tension trabeculae. The development process of trabeculae in Ward's triangle helps us understand the mechanism of proximal femoral fractures and provides a basis for biomimetic design of new internal fixation of proximal femur.

## Conflict of Interest Statement

All authors have read and contributed to the submitted manuscript and have no conflict of interest to declare.

## Ethics Statement

This study was approved by the ethics committee of the Third Hospital of Hebei Medical University. Informed consent was obtained from all the participants.

## Author Contributions

YZ, WC and QZ designed the study. QZ, JL, HD and YZ searched relevant studies. KD, WY, JZ, HW, CR and WY analyzed and interpreted the data. KD wrote the manuscript. WC and YZ contributed most in the revision of this manuscript. All authors approved the final version of the manuscript.

## Funding Information

This study was supported by the Support Program for the National Natural Science Foundation of China (Grant No. 82072447, 82272578), the Hebei National Science Foundation‐Outstanding Youth Foundation (Grant No. H2021206329). The funding source has no role in study design, conduction, data collection or statistical analysis.

## Consent of publication

Written informed consent was obtained from patients/guardians to authorize the publication of their data.
